# TGFβ2-Hepcidin Feed-Forward Loop in the Trabecular Meshwork Implicates Iron in Glaucomatous Pathology

**DOI:** 10.1167/iovs.61.3.24

**Published:** 2020-03-17

**Authors:** Ajay Ashok, Suman Chaudhary, Alexander E. Kritikos, Min H. Kang, Dallas McDonald, Douglas J. Rhee, Neena Singh

**Affiliations:** 1 Department of Pathology, School of Medicine, Case Western Reserve University, Cleveland, Ohio, United States; 2 Department of Ophthalmology, School of Medicine, Case Western Reserve University, Cleveland, Ohio, United States

**Keywords:** hepcidin, TGF-β2, iron, ROS, glaucoma, trabecular meshwork

## Abstract

**Purpose:**

Elevated levels of transforming-growth-factor (TGF)-β2 in the trabecular meshwork (TM) and aqueous humor are associated with primary open-angle glaucoma (POAG). The underlying mechanism includes alteration of extracellular matrix homeostasis through Smad-dependent and independent signaling. Smad4, an essential co-Smad, upregulates hepcidin, the master regulator of iron homeostasis. Here, we explored whether TGF-β2 upregulates hepcidin, implicating iron in the pathogenesis of POAG.

**Methods:**

Primary human TM cells and human and bovine ex vivo anterior segment organ cultures were exposed to bioactive TGF-β2, hepcidin, heparin (a hepcidin antagonist), or N-acetyl carnosine (an antioxidant), and the change in the expression of hepcidin, ferroportin, ferritin, and TGF-β2 was evaluated by semiquantitative RT-PCR, Western blotting, and immunohistochemistry. Increase in reactive oxygen species (ROS) was quantified with dihydroethidium, an ROS-sensitive dye.

**Results:**

Primary human TM cells and bovine TM tissue synthesize hepcidin locally, which is upregulated by bioactive TGF-β2. Hepcidin downregulates ferroportin, its downstream target, increasing ferritin and iron-catalyzed ROS. This causes reciprocal upregulation of TGF-β2 at the transcriptional and translational levels. Heparin downregulates hepcidin, and reduces TGF-β2-mediated increase in ferritin and ROS. Notably, both heparin and N-acetyl carnosine reduce TGF-β2-mediated reciprocal upregulation of TGF-β2.

**Conclusions:**

The above observations suggest that TGF-β2 and hepcidin form a self-sustained feed-forward loop through iron-catalyzed ROS. This loop is partially disrupted by a hepcidin antagonist and an anti-oxidant, implicating iron and ROS in TGF-β2-mediated POAG. We propose that modification of currently available hepcidin antagonists for ocular use may prove beneficial for the therapeutic management of TGF-β2-associated POAG.

TGF-β2 is implicated in the pathogenesis of primary open-angle glaucoma (POAG), one of the leading causes of blindness.^1^ Cumulative evidence suggests that aberrant signaling of TGF-β2 alters extracellular matrix (ECM) homeostasis by inducing cross-linking of actin networks and increased synthesis and deposition of fibrillogenic proteins in the trabecular meshwork (TM), interfering with its pliability and response to mechanical stress. This increases resistance to aqueous humor (AH) outflow and elevates intraocular pressure (IOP), a precipitating factor of glaucomatous pathology.^2^^–^^5^ Thus, pharmacological agents that reduce or neutralize bioactive TGF-β2 or block downstream pathways of TGF-β2 signaling are being explored as possible therapeutic options.^6^^–^^8^ Although partly successful, a better understanding of TGF-β2-mediated signaling and cross-talk with other pathways is necessary to improve the efficacy of existing agents and explore better options.

The principal downstream pathways of TGF-β2 include canonical or Smad-dependent, and noncanonical or Smad-independent signaling.^3^^,^^9^ Smad-dependent signaling involves association of phosphorylated Smad2 and 3 with co-Smad4, and translocation of the complex to the nucleus for transcriptional activation of fibrillogenic proteins that deposit in the ECM. Interestingly, Smad4 is a transcriptional activator of hepcidin,^10^ a hepatic peptide hormone that maintains serum iron within a narrow range by downregulating ferroportin (Fpn), the only known iron export protein.^11^^,^^12^

In addition to the liver, the heart, kidney, lungs, and other organs synthesize hepcidin locally, suggesting additional regulation of iron possibly through cross-talk with liver hepcidin.^13^ In the eye, hepcidin is synthesized locally in the neuroretina and ciliary epithelium, suggesting local regulation of iron transport to the neuroretina and the AH respectively.^14^^–^^16^ Expression of hepcidin in the corneal endothelium, lens epithelium, and TM indicate additional regulation of iron exchange between these structures and the AH.^15^ Although helpful in maintaining stringent regulation of iron, hepcidin is upregulated by inflammatory cytokines as well,^17^^–^^19^ and is likely to create a potentially toxic microenvironment by increasing intracellular iron and iron-catalyzed reactive oxygen species (ROS).^20^ In this respect, it is notable that TM cells respond to mechanical stress by upregulating TGF-β1 and IL-6,^21^ cytokines known to upregulate hepcidin,^17^^,^^22^^,^^23^ and the limited but noticeable protection of retinal ganglion cells by iron chelators suggests a prominent role of iron in the pathogenesis of POAG.^24^ Here, we explored whether local hepcidin in the TM is upregulated by TGF-β2, and the role of downstream pathways of hepcidin activation in TGF-β2-associated POAG.

## Methods

### Experimental Design

Primary human TM cells, human and bovine TM tissue, ex vivo human and bovine whole globe, anterior segment perfusion culture, and anterior cup models from cadaveric human and bovine eyes were used to evaluate the effect of TGF-β2 on hepcidin and vice versa in the TM. For primary human TM cells, recombinant bioactive TGF-β2 peptides or recombinant hepcidin peptides were used as triggers. For experiments with TM tissue, human and bovine anterior segment samples were infused, or cultured with replication-defective adenovirus expressing bioactive TGF-β2 (AdhuTGF-β2) or vector (AdEmpty).^25^^–^^27^ Ferric ammonium citrate (FAC) was used as a positive control to trigger hepcidin upregulation,^12^ heparin as a hepcidin antagonist,^28^^–^^30^ and N-acetyl-L-Carnosine^31^ as an antioxidant in different experimental paradigms.

### Human and Bovine Eye Globes

Human eye globes were acquired from the Lions Gift of Sight eye bank (St. Paul, MN, USA). Donors ranged in age from 29 to 92 years (Supplementary Table S1). All experiments involving human tissue and cells were performed in compliance with the tenets of the Declaration of Helsinki. Bovine eyes were collected from a local abattoir within 2 hours of euthanization.

### Isolation and Culture of Human and Bovine TM Tissue and Cells

TM cells were isolated from human eye globes as described,^32^ and cultured in DMEM supplemented with 1% FBS. Cultures from passages 2 and 3 at a confluence of 80% to 90% were used for all experiments. Each culture was tested for dexamethasone-induced upregulation of myocilin before use.^32^^,^^33^

### Ex Vivo Perfusion of Human Anterior Segment

Human anterior segment ex vivo perfusion culture was established as described^25^^–^^27^ (Supplementary Table S1), and perfused with DMEM containing 1% FBS at a constant flow rate of 2.5 µl/min using micro infusion pumps (Harvard Apparatus, Holliston, MA, USA). After stabilizing the pressure for 12 hours, control and experimental samples were infused intracamerally with AdEmpty or AdhuTGFβ2, respectively.^26^ The change in pressure was recorded with a computerized system (National Instruments, Austin, TX, USA) and plotted relative to time. Finally, the samples were collected, rinsed, fixed, and processed for immunohistochemistry.

### Ex Vivo Culture Model of Bovine Anterior Eye Cup and Whole Globe

The bovine anterior eye cup was cultured as described.^34^^,^^35^ In short, all tissues were removed from the anterior cup except the TM and cornea, and immersed in DMEM supplemented with 1% FBS and 1% penicillin/streptomycin at 37°C in a humidified atmosphere with 5% CO_2_. After overnight equilibration, samples were subjected to different experimental conditions, and the TM was isolated and analyzed. For experiments with the whole globes, AH was replaced with an equal volume of DMEM as above, supplemented with vehicle or FAC, and agitated gently in a CO_2_ incubator for 2 hours.^36^ Finally, control and experimental globes were dissected to isolate the TM.

### Chemicals

Dexamethasone (D1756), dihydroethidium (DHE), FAC (F5879), human recombinant TGF-β2 (T2815), heparin (H3149), and H_2_O_2_ (H1009) were from Sigma Aldrich, USA. Human hepcidin-25 peptide (4040671) and trifluoroacetic acid were from BACHEM, USA. Reconstitution Buffer-4 was from R&D System, USA. Fluoromount-G (0100-01) was from SouthernBiotech, USA, and N-acetyl-L-Carnosine (18817) was from Cayman Chemicals, USA.

### Immunohistochemistry

Immunohistochemistry was performed as described^37^ (antibody list in Supplementary Table S2). Stained sections were mounted and imaged with Leica inverted microscope (DMi8).

### ROS Detection

Release of superoxide anion was analyzed with the fluorescent probe DHE as described.^38^

### SDS-PAGE, Western Blotting, and RT-PCR

Protein lysates were fractionated by SDS-PAGE and analyzed by Western blotting as described.^15^^,^^16^^,^^33^^,^^37^ Proteins of interest were visualized by probing with specific antibodies (antibody list in Supplementary Table S2). RT-PCR was carried out as described earlier^15^ (primer list in Supplementary Table S3).

### Statistical Analysis

Densitometry of images was performed with UN-SCAN-IT gels (version 6.1) software (Silk Scientific, USA) and Image J software. All data were statistically analyzed by GraphPad Prism (version 5.0) software (GraphPad Software Inc., USA), and are shown as mean ± SEM. Significant differences between control and experimental samples were determined by the Student’s unpaired *t*-test or 2-way ANOVA. Differences were considered statistically significant starting at *P* < 0.05.

## Results

### Hepcidin is Synthesized in the Trabecular Meshwork and Upregulated by Iron

**Figure 1.**
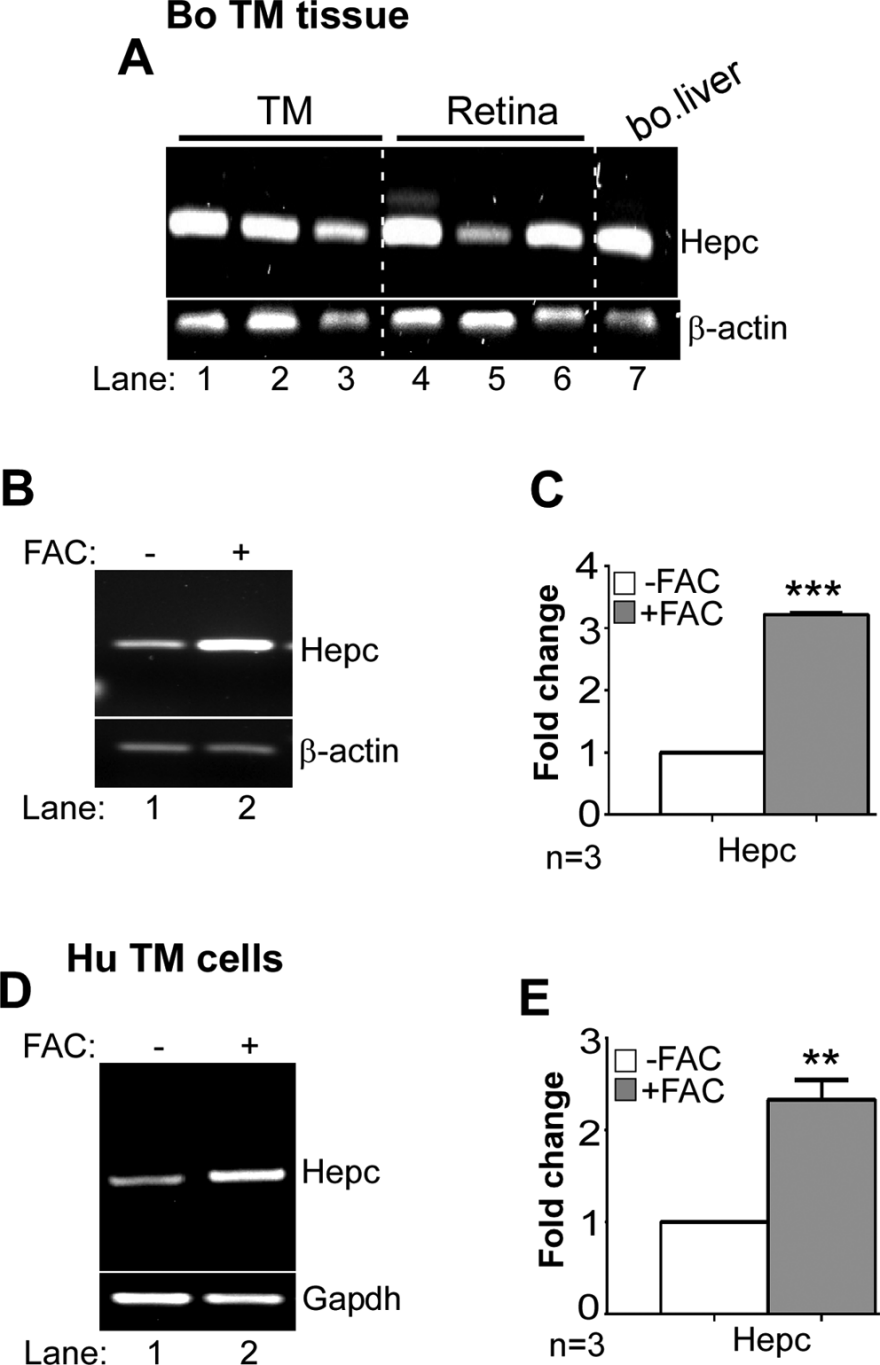
Synthesis of local hepcidin in bovine and human TM. (**A**) Amplification of hepcidin from bovine TM and retina by RT-PCR shows a band that co-migrates with hepcidin from bovine liver (lanes 1−7). β-Actin was amplified from the same samples for semiquantitative estimation. Full images are shown in Supplementary Figure S1-1A. (**B**) Amplification of hepcidin from bovine TM exposed to FAC or vehicle by RT-PCR shows upregulation of hepcidin by FAC relative to control (lanes 1 & 2). Full images are shown in Supplementary Figure S1-1B. (**C**) Densitometry after normalization with β-actin shows three-fold upregulation of hepcidin by FAC relative to controls. Values are mean ± SEM of the indicated n. ***P < 0.001. (**D**) Amplification of hepcidin from primary human TM cells exposed to FAC or vehicle by RT-PCR shows upregulation of hepcidin by FAC relative to control (lanes 1 & 2). Full images are shown in Supplementary Figure S1-1D. (**E**) Densitometry after normalization with GAPDH shows 2.3-fold upregulation of hepcidin by FAC relative to controls. Values are mean ± SEM of the indicated n. **P < 0.01.

Local synthesis of hepcidin in the TM was determined by subjecting freshly harvested bovine tissue from three eyes to semiquantitative RT-PCR with hepcidin-specific primers. Tissue from bovine retina and liver were analyzed in parallel as positive controls (Fig. 1A). Remarkably, a band co-migrating with retinal and liver hepcidin was amplified from all three samples (Fig. 1A, lanes 1−3 vs. 4−7). Amplified bands were eluted and sequenced to confirm their identity (Supplementary Fig. S2). The β-actin amplified in parallel indicated variable expression of hepcidin in TM and retina samples, although noticeably less than the liver (Fig. 1A, lanes 1−6 vs. 7).

To evaluate whether hepcidin in the TM responds to exogenous iron as expected, AH from three bovine eye globes was replaced with equal volume of medium containing 1% FBS supplemented with 30 µM FAC or vehicle, and the globes were agitated gently for 2 hours at 37°C. Subsequently, TM was harvested and analyzed. Semiquantitative RT-PCR revealed significant upregulation of hepcidin in samples exposed to FAC relative to controls (Fig. 1B, lanes 1,2; Fig. 1C).

To confirm the above results, primary human TM cells were cultured in the presence of 30 µM of FAC or vehicle for 24 hours, and analyzed as above. Exposure to FAC resulted in significant upregulation of hepcidin relative to controls (Fig. 1D, lanes 1,2; Fig. 1E), supporting the results in bovine TM tissue.

Together, the above results demonstrate local synthesis of hepcidin in human and bovine TM, and transcriptional upregulation by excess iron in the local microenvironment. Subsequent experiments were aimed at evaluating the cross-talk between TGF-β2 and hepcidin, and downstream pathways of hepcidin-mediated regulation of iron in the TM.

### TGF-β2 Upregulates Hepcidin and Downstream Proteins in the Trabecular Meshwork

**Figure 2.**
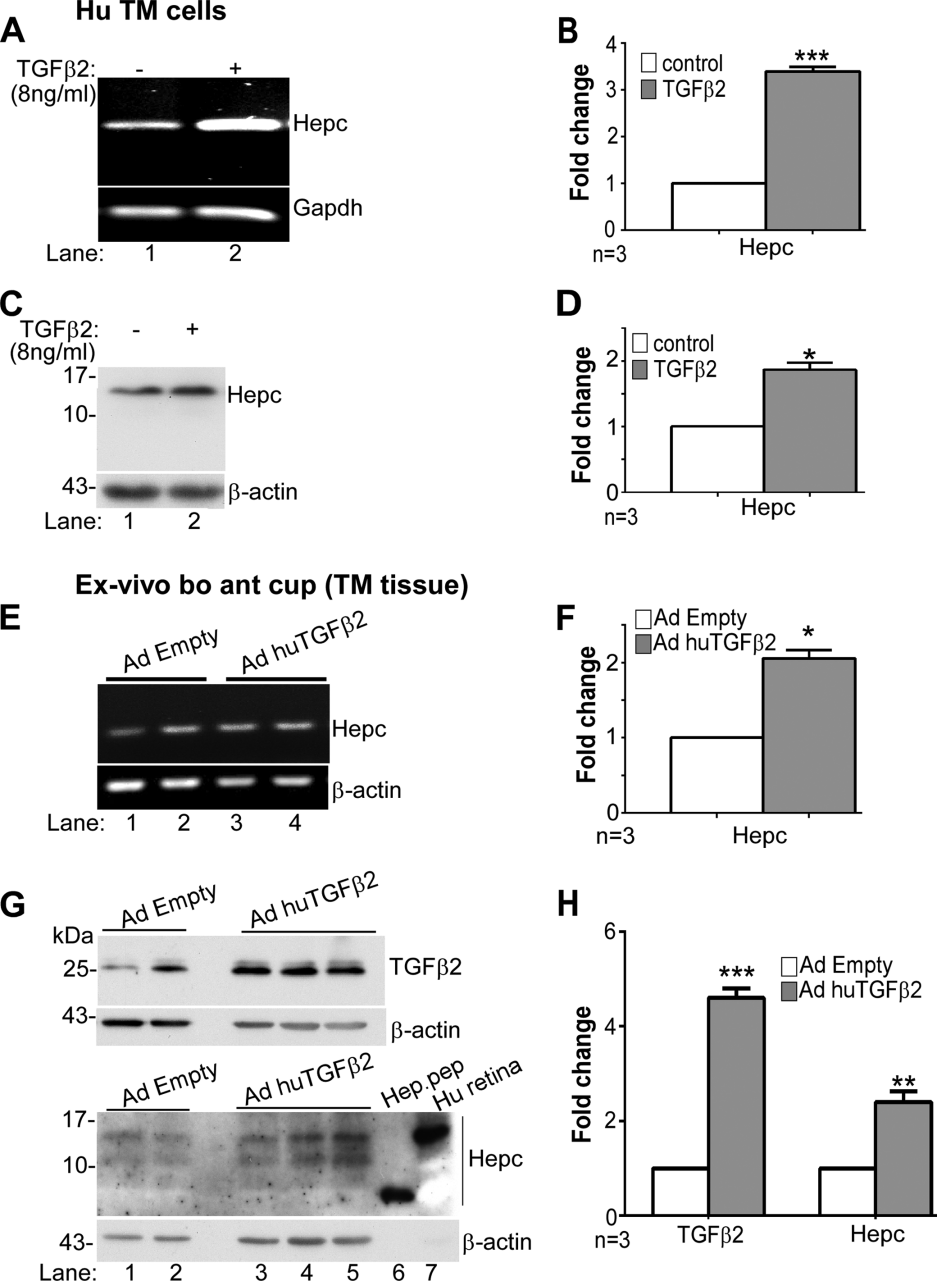
TGF-β2 upregulates hepcidin in the TM. (**A**) Amplification of hepcidin from primary human TM cells exposed to TGF-β2 or vehicle shows upregulation of hepcidin by TGF-β2 relative to the control (lanes 1 & 2). Full images are shown in Supplementary Figure S1-2A. (**B**) Densitometry after normalization with GAPDH shows 3.4-fold upregulation of hepcidin by TGF-β2 relative to controls. Values are mean ± SEM of the indicated n. ***P < 0.001. (**C**) Western blotting of lysates from primary human TM cells exposed to TGF-β2 or vehicle followed by probing for hepcidin shows increased expression of pro-hepcidin (approximately 12 kDa) by TGF-β2 relative to the control (lanes 1 & 2). Full images are shown in Supplementary Figure S1-2C. (**D**) Densitometry after normalization with β-actin shows 1.75-fold upregulation of pro-hepcidin by TGF-β2 relative to controls (lanes 1 & 2). Values are mean ± SEM of the indicated n. *P < 0.05. (**E**) Amplification of hepcidin from bovine TM overexpressing virally encoded human TGF-β2 (AdhuTGF-β2) shows increased expression relative to controls expressing empty vector (AdEmpty) (lanes 3 & 4 vs. 1 & 2). Full images are shown in Supplementary Figure S1-2E. (**F**) Densitometry after normalization with β-actin shows two-fold upregulation of hepcidin by TGF-β2 relative to controls. Values are mean ± SEM of the indicated n. *P < 0.05. (**G**) Western blotting of lysates from TM tissue in **E** above followed by probing for TGF-β2 shows increased expression of bioactive TGF-β2 in samples overexpressing TGF-β2 relative to controls as expected (lanes 3−5 vs. 1 & 2). Reaction for β-actin provides a loading control. Western blot analysis of the same lysates for hepcidin shows increased expression of pro-hepcidin in samples overexpressing TGF-β2 relative to controls (lanes 3−5 vs. 1 & 2). Recombinant hepcidin peptide and lysate from human retina were fractionated as positive controls, and showed hepcidin reactive bands at 3 kDa and approximately 12 kDa respectively (lanes 6 & 7). The membrane was re-probed for β-actin as a loading control. Full images are shown in Supplementary Figure S1-2G. (**H**) Densitometry after normalization with β-actin shows 4.3-fold increase in TGF-β2 and 2.2-fold increase in hepcidin in samples over-expressing TGF-β2 relative to controls. Values are mean ± SEM of the indicated n. **P < 0.01; ***P < 0.001.

To evaluate whether TGF-β2 upregulates hepcidin, two experimental models were tested; primary human TM cells exposed to recombinant bioactive TGF-β2 peptide at a concentration of 4 to 8 ng/mL diluted in reconstitution buffer-4 (Figs. 2A−D), and ex vivo bovine anterior eye cup model overexpressing human bioactive TGF-β2 (Figs. 2E−H). The dose of TGF-β2, although higher than pathological levels observed in POAG, is within the range that upregulates fibrillogenic proteins in vitro in primary human TM cells^5^^,^^25^^,^^39^^,^^40^ (Supplementary Fig. S3).

Thus, human TM cells were exposed to 8 ng/mL of TGF-β2 or vehicle for 24 hours, and analyzed. Amplification of hepcidin by RT-PCR revealed significant upregulation by TGF-β2 relative to controls (Fig. 2A, lanes 1,2; Fig. 2B). Western blotting and probing of protein lysates for hepcidin mirrored the above results, and showed significant upregulation of pro-hepcidin migrating at approximately 12 kDa^41^^,^^42^ by TGF-β2 relative to controls (Fig. 2C, lanes 1,2; Fig. 2D). The membrane was re-probed for β-actin as a loading control.

To confirm the above results in TM tissue, ex vivo culture of bovine anterior eye cup was infected with replication defective adenovirus expressing bioactive human TGF-β2 (AdhuTGF-β2).^26^ Control samples received a similar titer of virus expressing empty vector (AdEmpty). Following an incubation of 2 hours, TM was isolated and processed. Amplification of bovine hepcidin by RT-PCR showed significant upregulation in samples overexpressing TGF-β2 relative to controls (Fig. 2E, lanes 3,4 vs. 1,2; Fig. 2F). Western blotting of protein lysates from the same samples and probing for TGF-β2^43^ confirmed significant increase in expression by AdhuTGF-β2 within 2 hours relative to controls (Fig. 2G upper panel, lanes 3−5 vs. 1,2; Fig. 2H). Re-probing for β-actin provided a loading control. A similar analysis for hepcidin showed significant upregulation in samples overexpressing TGF-β2 relative to vector controls (Fig. 2G lower panel, lanes 3−5 vs. 1,2; Fig. 2H). Recombinant hepcidin peptide migrated at approximately 3 kDa as expected, and samples from human retina showed an approximately 12 kDa band of pro-hepcidin co-migrating with hepcidin from the TM (Fig. 2G lower panel, lanes 6,7; Fig. 2H).^44^^,^^45^ The membrane was re-probed for β-actin as a loading control.

**Figure 3.**
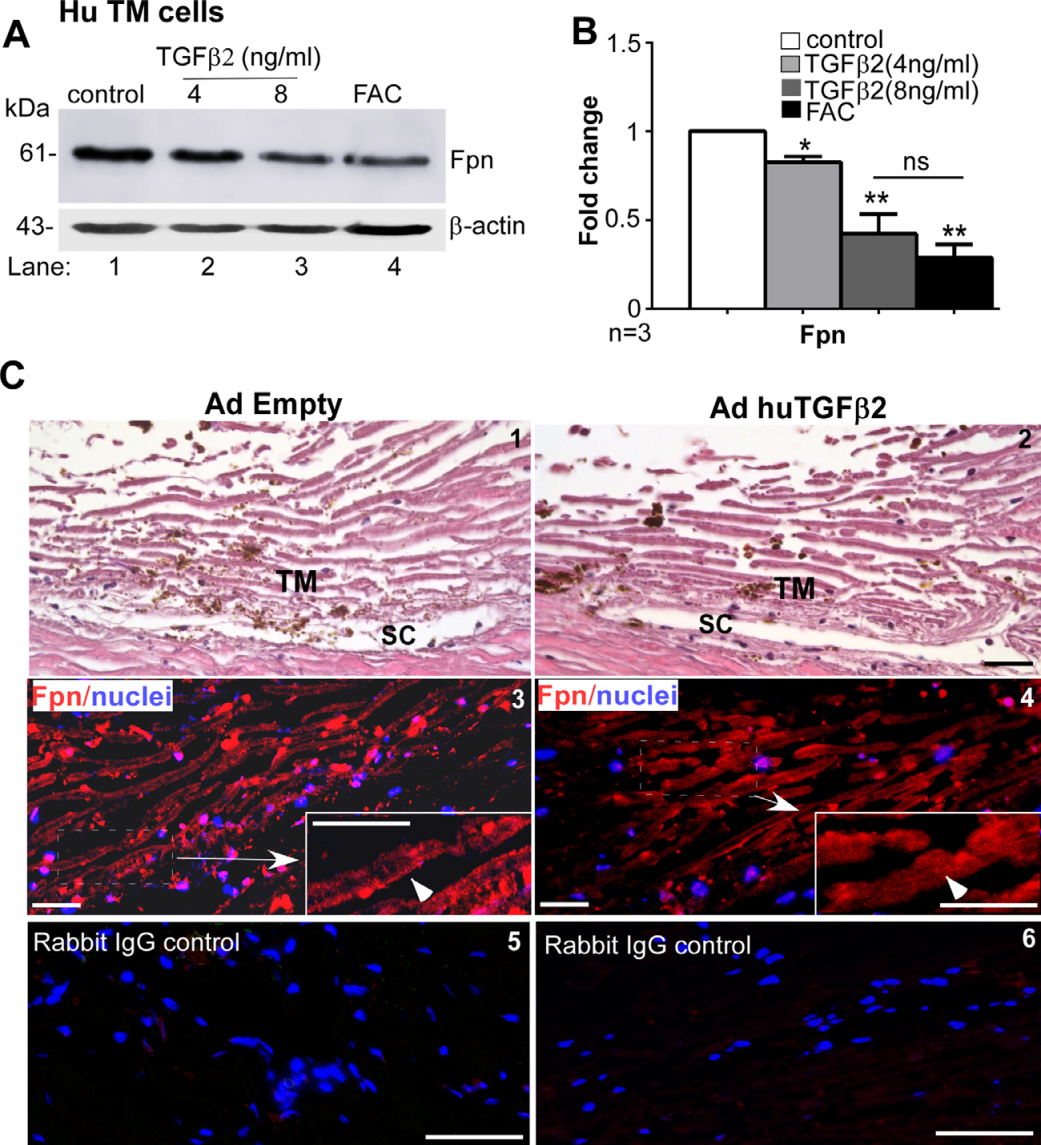
TGF-β2 downregulates ferroportin in human TM. (**A**) Western blotting of lysates from primary human TM cells exposed to TGF-β2 or FAC shows decrease in Fpn expression relative to control (lanes 2−4 vs. 1). Full images are shown in Supplementary Figure S1-3A. (**B**) Densitometry after normalization with β-actin shows a dose dependent decrease in Fpn of 1.2-fold and 2.3-fold with 4 and 8 ng/mL of TGF-β2, respectively, relative to controls. The decrease in Fpn by FAC is equivalent to 8 ng/mL of TGF-β2. Values are mean ± SEM of the indicated n. *P < 0.05, **P< 0 .01. (**C**) Hematoxylin and eosin staining of fixed tissue from human ex vivo anterior segment model of POAG overexpressing TGF-β2 shows the expected architecture of TM and the Schlemm's canal (SC) (panels 1 & 2). Immunoreaction of serial sections for Fpn shows expression on the plasma membrane in the control sample (panel 3, arrowhead), and internalization and downregulation in TGF-β2-expressing sample that showed increase in IOP with time (panel 4, arrowhead) (Supplementary Figure S4). Serial sections exposed to rabbit IgG and processed in parallel shows no reactivity (panels 5 & 6). Scale bar: 25 µm.

Because hepcidin regulates iron by downregulating Fpn, downstream effects of TGF-β2-mediated upregulation of hepcidin were evaluated by exposing primary human TM cells to 4 and 8 ng/mL of TGF-β2 for 24 hours, and analysis of protein lysates by Western blotting. Parallel cultures were exposed to 30 µM of FAC as a positive control. Probing for Fpn revealed dose-dependent decrease in Fpn by TGF-β2, the effect of 8 ng/mL similar to FAC (Fig. 3A, lanes 2−4 vs. 1; Fig. 3B).

Relevance of the above results to POAG was explored in the ex vivo human anterior segment culture model of glaucoma overexpressing TGF-β2 in the TM.^25^ Following overnight equilibration, the samples were infused with AdhuTGF-β2 or vector, and change in pressure was monitored over time. As expected, there was a gradual but significant increase in pressure in TGF-β2-expressing sample relative to the control after 6 days (Supplementary Fig. S4), at which time the samples were removed and processed for immunohistochemistry. Staining with hematoxylin and eosin (H & E) confirmed preservation of architecture in both samples with clear demarcation of TM and Schlemm's canal (Fig. 3C, panels 1,2). Immunostaining of serial sections for Fpn showed internalization and downregulation of Fpn in TM cells overexpressing TGF-β2 relative to vector control (Fig. 3C, panels 3,4). Incubation of serial sections with rabbit IgG did not show any reaction, confirming the specificity of Fpn reactivity (Fig. 3C, panels 5,6).

**Figure 4.**
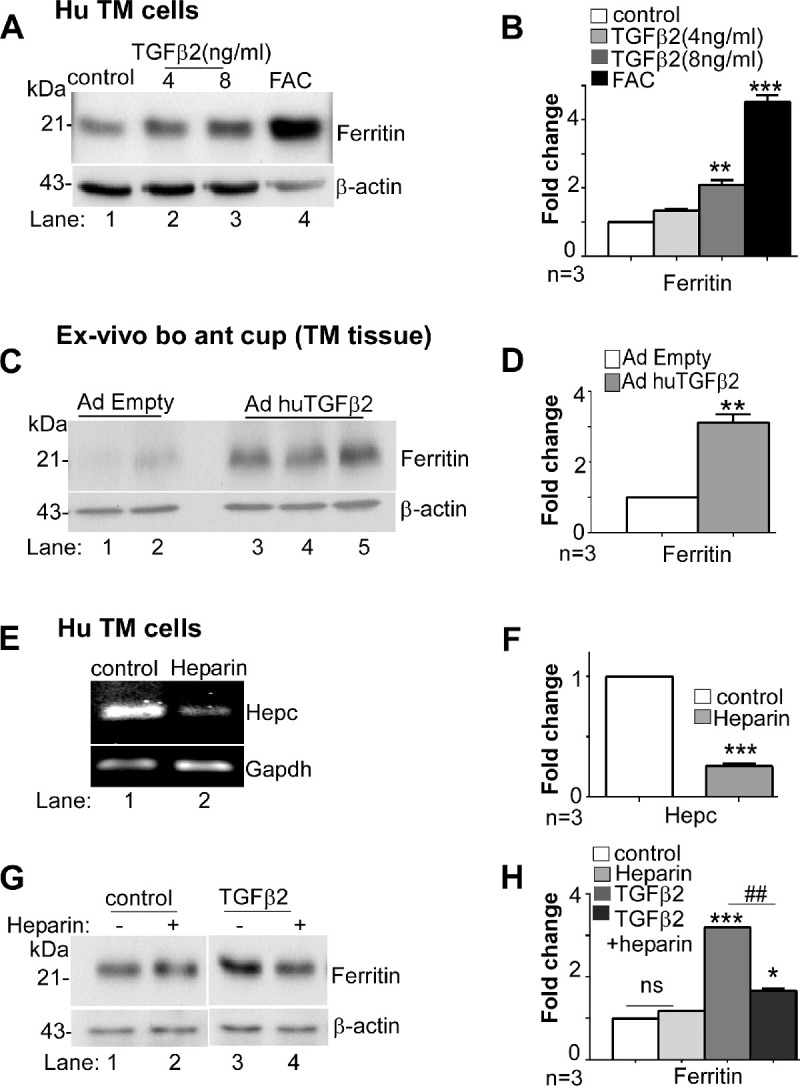
TGF-β2 upregulates ferritin in human TM. (**A**) Western blotting of lysates from primary human TM cells exposed to TGF-β2 or FAC shows increased expression of ferritin by TGF-β2 and FAC relative to vehicle treated control (lanes 2−4 vs. 1). Full images are shown in Supplementary Figure S1-4A. (**B**) Densitometry after normalization with β-actin shows two-fold upregulation of ferritin by 8 ng/mL TGF-β2 and 4.2-fold increase by FAC. Values are mean ± SEM of the indicated n. **P < 0.01. (**C**) Immunoblotting of TM tissue from bovine *ex*-*vivo* anterior eye cup overexpressing human TGF-β2 shows increased expression of ferritin relative to vector expressing control (lanes 3−5 vs. 1 & 2). Full images are shown in Supplementary Figure S1-4C. (**D**) Quantification by densitometry after normalization with β-actin shows three-fold increase in ferritin by TGF-β2 relative to controls. Values are mean ± SEM of the indicated n. **P < 0.01. (**E**) Treatment of primary human TM cells with heparin followed by amplification of hepcidin by RT-PCR shows significant downregulation relative to vehicle treated control (lane 2 vs. 1). Full images are shown in Supplementary Figure S1-4E. (**F**) Densitometry after normalization with GAPDH shows reduction in hepcidin mRNA by heparin to one-fifth of the control value. Values are mean ± SEM of the indicated n. ***P < 0.001. (**G**) Western blotting of lysates from primary human TM cells exposed to TGF-β2 in the absence or presence of heparin shows upregulation of ferritin in the absence of heparin (lane 3 vs. 1) and significant reduction by pre-incubation with heparin (lane 4 vs. 3). Vehicle or heparin treated controls did not show any change (lanes 1 & 2). Full images are shown in Supplementary Figure S1-4G. (**H**) Densitometry after normalization with β-actin shows three-fold upregulation of ferritin by TGF-β2, and a reduction to ½ the control value with heparin. Values are mean ± SEM of the indicated n. *P < 0.05; ***P < 0.001; ^##^*P* < 0.01 (*TGF-β2 vs. control; # TGF-β2 without and with heparin).

To evaluate whether the TGF-β2-mediated downregulation of Fpn through hepcidin increases intracellular iron and upregulates ferritin, primary human TM cells were exposed to 4 and 8 ng/mL of TGF-β2 for 24 hours as above, and lysates were processed for Western blotting. Probing for ferritin showed significant upregulation in cells exposed to 8 ng/mL of TGF-β2 relative to controls (Fig. 4A, lanes 2,3 vs. 1; Fig. 4B), although less than FAC (Fig. 4A, lane 4; Fig. 4B). Downregulation of Fpn by siRNA also upregulated ferritin, confirming the hepcidin-Fpn-iron axis in these cells (Supplementary Fig. S5).

A similar evaluation in bovine ex vivo anterior eye cup model infected with AdhuTGF-β2 for 2 hours showed significant upregulation of ferritin relative to vector controls (Fig. 4C, lanes 3−5 vs. 1,2; Fig. 4D). Immunostaining of fixed sections, likewise, showed relatively stronger reactivity for ferritin in TM sections infected with AdhuTGF-β2 relative to AdEmpty controls (Supplementary Fig. 7, panels 1−4). Serial sections reacted with rabbit IgG control showed negative results, confirming the specificity of the reaction (Supplementary Fig. S7, panel 5,6).

To evaluate the specific role of hepcidin in TGF-β2-mediated upregulation of ferritin, primary human TM cells were pre-incubated with heparin (200 µg/mL) for 16 hours, a hepcidin antagonist,^29^^,^^30^ before the addition of 8 ng/mL of TGF-β2 for an additional 24 hours. This dose of heparin did not induce toxicity in primary human TM cells (Supplementary Fig. S6) or influence ferritin levels (Fig. 4G, lanes 1,2; Fig. 4H). Downregulation of hepcidin by heparin was evident when assessed by RT-PCR with hepcidin-specific primers (Fig. 4E, lanes 1,2; Fig. 4F). Evaluation of protein lysates from heparin pretreated samples exposed to TGF-β2 by Western blotting showed significant downregulation of ferritin by heparin relative to untreated controls (Fig. 4G, lane 4 vs. 3; Fig. 4H). These results indicate a prominent role of hepcidin in TGF-β2-mediated upregulation of ferritin.

### Reciprocal Upregulation of TGF-β2 by Hepcidin Through ROS

To evaluate whether TGF-β2 increases ROS through hepcidin, triplicate cultures of primary human TM cells were pretreated with 200 µg/mL of heparin for 16 hours followed by 8 ng/mL of TGF-β2 for an additional 24 hours. Additional cultures were incubated with medium supplemented with 0.1% trifluoroacetic acid or 200 µg/mL of heparin as negative controls, and 1 µg/mL of recombinant hepcidin diluted in 0.1% trifluoroacetic acid or 30 µM of FAC as positive controls. One set of cultures were treated with 0.2 mM of H_2_O_2_ for the entire duration of the experiment, and considered as a positive control at 100% ROS (standardized in primary human TM cells; Supplementary Fig. S8). None of the treatments exhibited significant cytotoxicity as estimated by the lactate dehydrogenase (LDH) assay (Supplementary Fig. S9).

**Figure 5.**
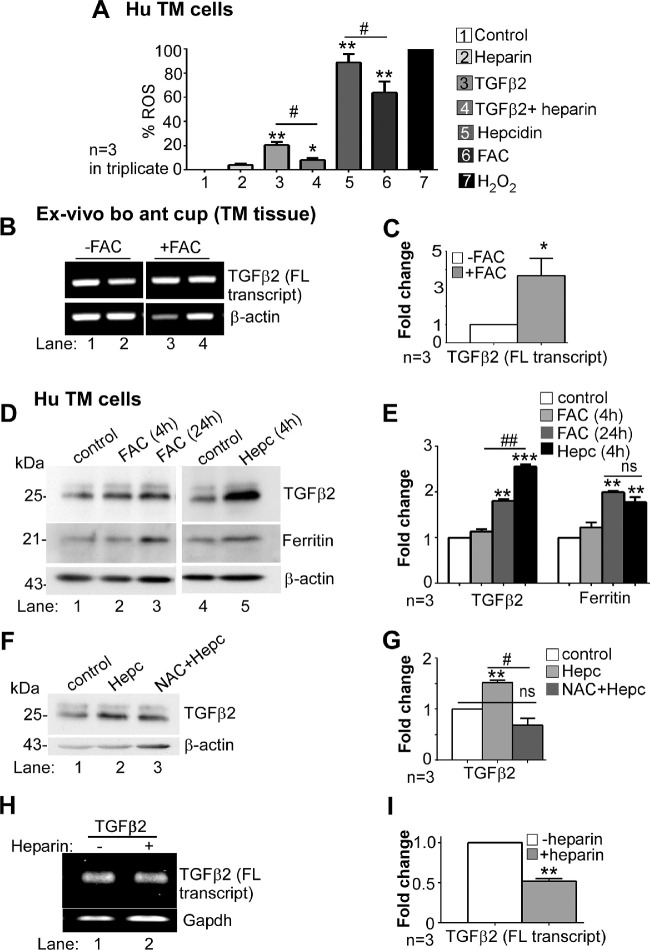
Iron and ROS upregulate TGFβ2. (**A**) Intracellular ROS in primary human TM cells was quantified by recording the change in fluorescence intensity of DHE in triplicate cultures exposed to medium (0%), heparin, TGF-β2, TGF-β2 with heparin, hepcidin, FAC, and 0.2 mM H_2_O_2_ (100%). Exposure to hepcidin showed maximum increase in ROS by 88%, followed by 63% by FAC, and 20% by TGF-β2. Pre-incubation with heparin reduced TGF-β2-induced ROS to 8%. Values are mean ± SEM of the indicated n. *P < 0.05, **P < 0.01; ^#^*P* < 0.05 (*treatment vs. control, ^#^TGFβ2 vs. heparin + TGFβ2). (**B**) Amplification of TGF-β2 by RT-PCR from TM tissue harvested from ex vivo bovine anterior eye cup exposed to FAC or vehicle shows significant upregulation TGF-β2 relative to controls (lanes 3 & 4 vs. 1 & 2). Full images are shown in Supplementary Figure S1-5B. (**C**) Quantification by densitometry shows 3.6-fold upregulation of TGF-β2 by FAC relative to controls. Values are mean ± SEM of the indicated n. *P < 0.05. (**D**) Exposure of primary human TM cells to FAC or hepcidin shows upregulation of TGF-β2 by 24-hour exposure to FAC (lane 1 vs. 3) and hepcidin relative to vehicle treated control (lane 5 vs. 4). Re-probing for ferritin shows increased expression by FAC (lane 3 vs. 1) and hepcidin (lane 5 vs. 4). Full images are shown in Supplementary Figure S1-5D. (**E**) Densitometry after normalization with β-actin shows 1.8-fold and 2.4-fold increase in TGF-β2, and 2-fold and 1.8-fold increase in ferritin by FAC (24 hours) and hepcidin respectively. Increase in TGF-β2 by hepcidin is significantly more than 24 hour exposure to FAC. Values are mean ± SEM of the indicated n. **P < 0.01, ***P < 0.001; ^##^*P* < 0.01 (*TGF-β2 vs. control; ^#^24 hours FAC vs. 4 hours hepcidin). (**F**) Primary human TM cells were exposed to hepcidin, or pre-incubated with NAC for 1 hour before adding hepcidin. Hepcidin induced significant upregulation of bioactive TGF-β2 (lane 2 vs. 1), which was reduced to control levels by NAC (lanes 1−3). Full images are shown in Supplementary Figure S1-5F. (**G**) Quantification by densitometry shows 1.5-fold upregulation of bioactive TGF-β2 by hepcidin relative to controls. Pre-treatment with NAC abolishes this effect. Values are mean ± SEM of the indicated n. **P < 0.01; ^#^*P* < 0.05 (^#^hepcidin vs. NAC + hepcidin). (**H**) Amplification of TGF-β2 by RT-PCR from primary human TM cells incubated with bioactive TGF-β2 in the absence or presence of heparin shows significant downregulation of full-length TGF-β2 transcript by heparin (lane 2 vs. 1). Full images are shown in Supplementary Figure S1-5H. (**I**) Quantification by densitometry shows reduction of TGF-β2 mRNA to ½ of the control by heparin. Values are mean ± SEM of the indicated n. **P < 0.01.

Control and treated cultures were incubated with DHE for 40 minutes, and fluorescence intensity as a measure of superoxide anion was quantified relative to medium (0%) and H_2_O_2_ (100%). TGF-β2 increased ROS to 20%, which was reduced by heparin to 8%. Hepcidin caused the maximum increase in ROS to 88%, followed by FAC at 63% (Fig. 5A). These results suggest that TGF-β2 upregulates ROS through hepcidin.

Reciprocal upregulation of certain members of the TGF-β family by iron-catalyzed ROS is known,^46^ and was evaluated in ex vivo organ cultures of bovine anterior eye cup exposed to 30 µM of FAC for 24 hours. Amplification of bovine TGF-β2 by RT-PCR showed significant upregulation by FAC relative to controls (Fig. 5B, lanes 3,4 vs. 1,2; Fig. 5C).

To evaluate whether primary human TM cells respond similarly to iron, cultures were exposed to 30 µM of FAC for 4 and 24 hours, or 1 µg/mL of recombinant hepcidin peptide for 4 hours. Control cultures received equal volume of diluent. At the indicated times, cell lysates were evaluated by Western blotting. Probing for TGF-β2 revealed significant upregulation of the 25 kDa bioactive form by exposure to FAC for 24 hours and hepcidin for 4 hours (Fig. 5D, lanes 2,3 vs. 1, and lane 5 vs. 4; Fig. 5E). Re-probing for ferritin showed significant increase by 24-hour exposure to FAC and 4 hour treatment with hepcidin relative to matched controls (Fig. 5D, lanes 2,3 vs. 1, and lane 5 vs. 4; Fig. 5E). Surprisingly, upregulation of TGF-β2 by hepcidin was significantly more than FAC although upregulation of ferritin was similar. These results suggest that increased generation of ROS by hepcidin relative to FAC noted in Figure 5A may be responsible for significantly more upregulation of TGF-β2 by the hepcidin.

To evaluate the role of ROS in hepcidin-mediated upregulation of TGF-β2, primary human TM cells were pre-incubated with the antioxidant, N-acetyl-L-Carnosine (NAC; 5 mM) for 1 hour^47^ before exposure to 1 µg/mL of recombinant hepcidin for 4 hours, and lysates were evaluated by Western blotting. Probing for TGF-β2 revealed significant upregulation of the 25 kDa bioactive form by hepcidin, which was reduced to control levels by pretreatment with NAC (Fig. 5F, lane 2 vs. 1, and lane 3 vs. 2; Fig. 5G).

The contribution of hepcidin in inducing reciprocal upregulation of TGF-β2 in primary human TM cells was evaluated by pre-incubating the cells with 200 µg/mL of heparin for 16 hours before exposure to 8 ng/mL of bioactive TGF-β2 for 24 hours. Amplification of TGF-β2 by RT-PCR showed significant downregulation by heparin treatment, suggesting that bioactive TGF-β2 induces transcriptional activation of TGF-β2 gene through hepcidin (Fig. 5H, lane 2 vs. 1; Fig. 5I).

**Figure 6.**
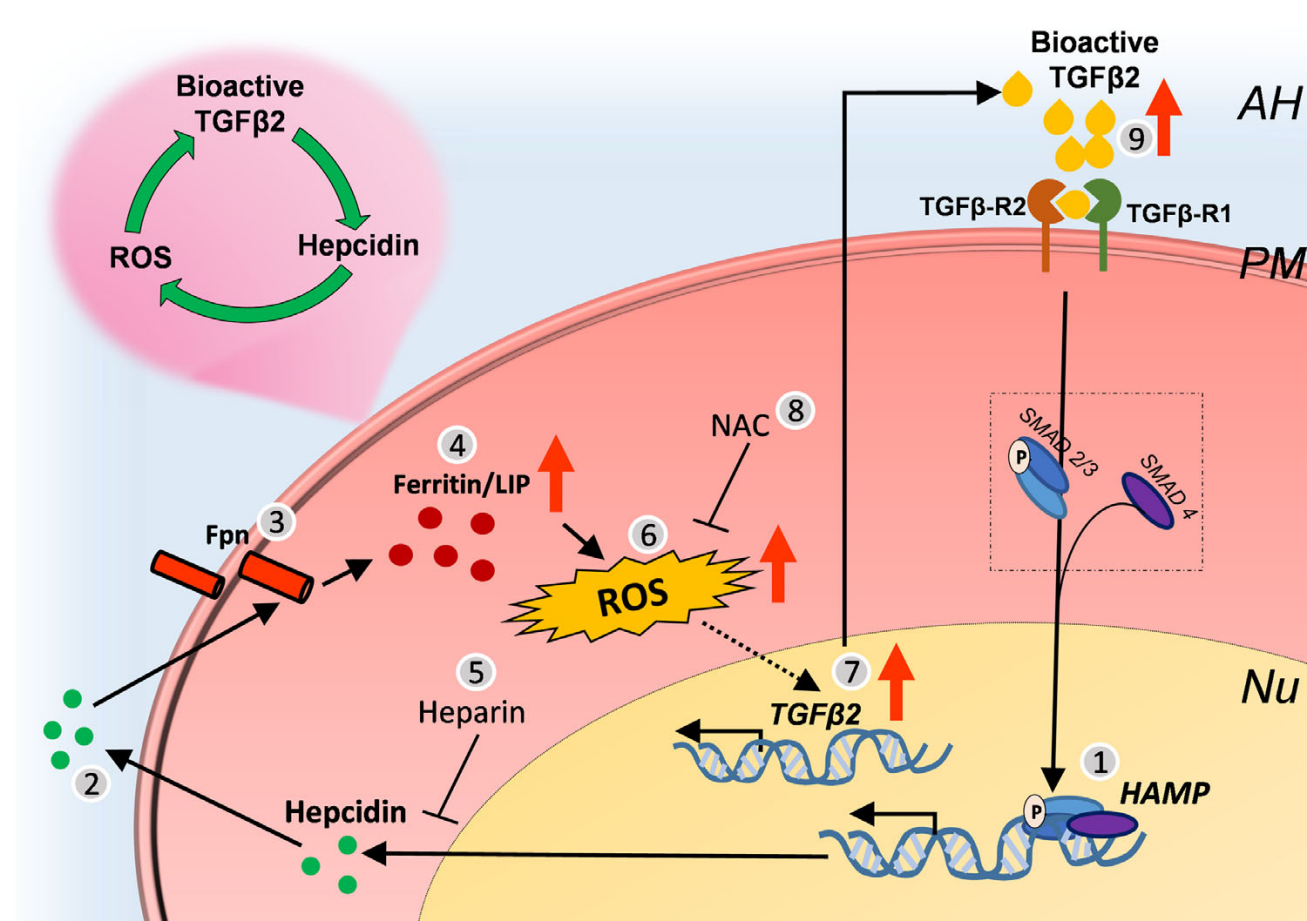
Graphical representation of the TGFβ2-Hepcidin feed-forward loop in the TM. (1) TGF-β2 upregulates transcription of hepcidin by the well-established Smad dependent (canonical) pathway^10^^,^^56^ (Figs. 2A−D; Figs. 2E−H), which (2) results in increased synthesis and secretion of hepcidin peptide. (3) Hepcidin (and TGF-β2) downregulate Fpn (Figs. 3A,B; Fig. 3C), resulting in (4) accumulation of iron in intracellular compartments and compensatory upregulation of ferritin (Figs. 4A-4 D. (5) Downregulation of hepcidin by heparin (Figs. 4E,4F) decreases TGF-β2-mediated upregulation of ferritin (Figs. 4G, 4H), implicating hepcidin in this process. (6) TGF-β2 increases ROS through hepcidin because heparin causes a signification reduction (Fig. 5A). (7) FAC and hepcidin also upregulate TGF-β2 (Figs. 5D,5E) and ferritin, suggesting a role of iron-catalyzed ROS in this process (Figs. 5D,5E). (8) Pre-incubation of TM cells with NAC decreases hepcidin-mediated upregulation of TGF-β2 (Figs. 5F,5G), implicating ROS in this process. (9) TGF-β2 triggers transcriptional activation of TGF-β2 (Figs. 5H,5I) and increased availability of bioactive TGF-β2, forming a self-sustained TGF-β2-hepcidin feed-forward loop.

Together, the above results suggest a self-sustained TGF-β2-hepcidin-TGF-β2 feed-forward loop through iron-catalyzed ROS, which is disrupted by the hepcidin antagonist heparin, and the anti-oxidant NAC (Fig. 6).

## Discussion

We report upregulation of local hepcidin in human and bovine TM tissue and primary human TM cells by TGF-β2, and reciprocal upregulation of TGF-β2 by hepcidin through iron-catalyzed ROS, forming a self-sustained positive feed-forward loop between TGF-β2 and hepcidin. These observations implicate iron-catalyzed ROS in TGF-β2-associated POAG, providing novel insight into the mechanistic basis of TGF-β2-associated POAG, and therapeutic options aimed at reducing hepcidin and iron-catalyzed ROS in the TM.

Local expression of hepcidin and Fpn in the TM re-inforce similar observations in a recent report,^15^ and suggest stringent regulation of iron at this site by its autocrine and paracrine activity.^11^^,^^12^^,^^48^^,^^49^ However, upregulation of hepcidin by TGF-β2 through Smad4, a potent transcriptional activator of hepcidin,^10^ is likely to increase intracellular iron and the potential for iron-catalyzed ROS. Our observations support this hypothesis, and indicate that TGF-β2 initiates a self-sustained feed-forward loop through hepcidin, implicating iron and ROS in TGF-β2-associated POAG.

As summarized in Figure 6, TGF-β2 upregulates hepcidin in TM cells possibly through Smad4, which downregulates Fpn in the same and adjacent cells by its autocrine and paracrine activity. This results in accumulation of iron in intracellular compartments. Although compensatory upregulation of ferritin stores iron in a relatively nonreactive form, chronic upregulation of hepcidin is likely to increase reactive oxygen and catalyze iron-mediated ROS, triggering the transcriptional activation of TGF-β2, and in addition, the bioactive form of TGF-β2 in the extracellular milieu. Interestingly, heparin, a hepcidin antagonist reduces TGF-β2-mediated increase in ferritin and ROS, and NAC reduces TGF-β2-mediated increase in ROS, supporting a role of hepcidin and ROS in this cycle. This is further supported by upregulation of TGF-β2 by FAC and hepcidin, implicating iron-catalyzed ROS as the proximate cause of increase in TGF-β2 levels. Moreover, bioactive TGF-β2 triggers transcriptional upregulation of TGF-β2, indicating that bioactive TGF-β2 initiates the hepcidin-TGF-β2 feed-forward loop, fueled by iron-catalyzed ROS.

A similar role of ROS in upregulating TGF-β isoforms has been described in other cell types^50^^–^^52^ and TM cells,^53^ supporting our observations. However, the pathways described above do not exist in isolation, and significant cross-talk with other biochemical pathways, some associated with POAG, is likely to influence the outcome in the in vivo conditions.

The significance of these observations is two-fold; first, this study demonstrates a clear involvement of iron-catalyzed ROS in TGF-β2-associated POAG, and second, the involvement of hepcidin in this process provides an untapped opportunity for using hepcidin antagonists as adjuncts to ROCK inhibitors and other treatment options for POAG.^4^ A range of hepcidin antagonists are currently undergoing clinical trials for systemic disorders,^54^^,^^55^ and could be modified for ophthalmic use.

## Supplementary Material

Supplement 1
