# Supplementary material for: TGFβ2-Hepcidin Feed-Forward Loop in the Trabecular Meshwork Implicates Iron in Glaucomatous Pathology
Source: Invest Ophthalmol Vis Sci. 2020 Mar 17;61(3):24. doi: 10.1167/iovs.61.3.24 (PMC7401420; doi:10.1167/iovs.61.3.24)
Supplement: Supplement 1 [file iovs-61-3-24_s001.pdf]

Supplementary file

Supplementary Table 1: List of biological subjects

Human tissues used for primary TM cell culture and whole tissue lysates<br>Source: Lions Gift of Sight			
**PMI (h)/ PCoD***	**Age (year)**	**Sex**	**Tissue region**
15/ Leukemia	68	M	Perfusion experiment
14 /Parkinson's Disease	92	F	Perfusion experiment
14/ Hodgkin's lymphoma	29	M	Perfusion experiment
14/Sepsis	77	F	Trabecular meshwork cell culture
3/end-stage renal disease	78	F	Trabecular meshwork cell culture
12/Multiple system failure	67	M	Trabecular meshwork cell culture
7.5/Acute cardiac event	65	M	Trabecular meshwork cell culture
			TM cells from Dr. Rhee's lab (from 6 different donors at passage 2).
**Bovine samples**			
**Breed**	**Tissue Region**		
Mixed	Trabecular meshwork/ Anterior Chamber/Whole globes		

***PMI (h)- Postmortem interval (from death till time of Enucleation) / PCoD- Primary cause of death**

Supplementary Table 2: List of antibodies

Primary Antibody	Host	Cat. No.	Company	Dilution
Hepcidin antimicrobial peptide	Rb	NBP1-59337	Novus biologicals, USA	WB- 7/1000
Ferroportin (Fpn)	Rb	NBP1-21502	Novus biologicals, USA	WB-2/500<br>IHC-1/100
TGFβ2	m	ab36495	abcam, USA	WB-1/500
Ferritin	Rb	F5012	Sigma Aldrich, USA	WB-1/1000<br>IHC-1/100
Vimentin	m	sc-6260	Santa Cruz, USA	WB-1/500
β-actin	m	MAB1501	Millipore, USA	WB-1/5000
Rabbit IgG, polyclonal - Isotype Control	-	ab37415	abcam, USA	IHC- 1/100
**Secondary Antibody**				
HRP-conjugated secondary anti- mouse	Sh	NA931V	GE Healthcare, USA	WB-1/10000
HRP-conjugated secondary anti- rabbit	Dn	NA934V	GE Healthcare, USA	WB-1/10000
Anti-Rabbit IgG (H+L) cross-adsorbed secondary antibody, Alexa Fluor 546	g	A11071	Invitrogen, USA	IHC- 1/1000

Rb- rabbit, m- mouse, Sh- sheep, Dn- donkey, g-goat; WB- Western blotting, IHC- immunohistochemistry

Supplementary Table 3: List of primers

Target gene	Sequence
bov hepcidin-F	5’-TCCTTGTCCTGCTCAGCCTG-3’
bov hepcidin-R	5’-CAGCAGAAGATGCAGATGGGA-3’
bov-TGFβ2-F	5’-TGGCTTCACCATAAAGACAGGA-3’
bov-TGFβ2-R	5’-TACAAAAGTGCAGCAGGGACA-3’
bov-β-ACTIN-F	5’-CTTCCTGGGCATGGAATCCT-3’
bov-β-ACTIN-R	5’-TTGATCTTCATTGTGCTGGGTG-3’
hu hepcidin-F	5’-CCTGACCAGTGGCTCTGTTT-3’
hu hepcidin-R	5’-CACATCCCACACTTTGATCG-3’
hu TGFβ2-F	5’-TGGCTTCACCATAAAGACAGGA-3’
hu TGFβ2-R	5’-TACAAAAGTGCAGCAGGGAC-3’
hu GAPDH-F	5’-GAGTCAACGGATTTGGTCGT-3’
hu GAPDH-R	5’-GGTGCCATGGAATTTGCCAT-3’

Supplementary Figure 1 (S1)

Fig S1. Full images for all cropped images are presented.

Fig. 1 in manuscript

Figure 1.A

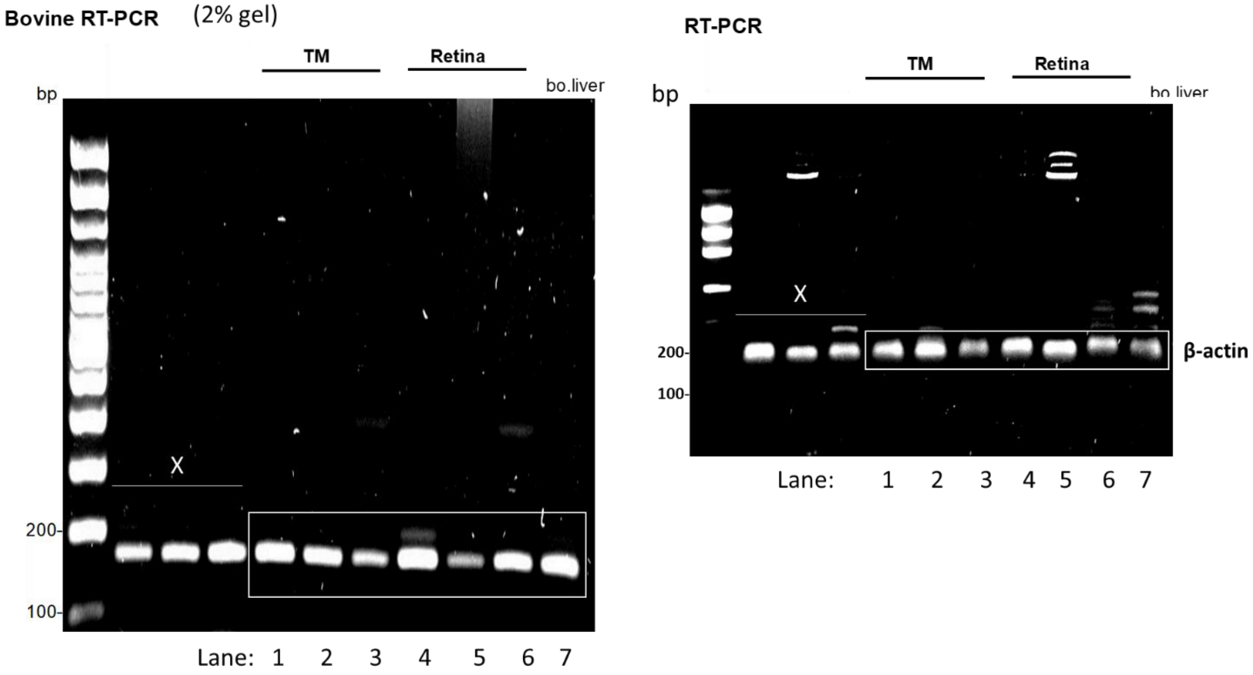

Figure 1.B

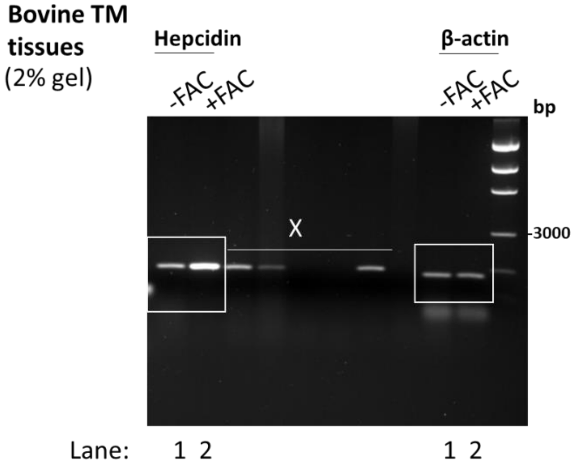

Figure 1.D

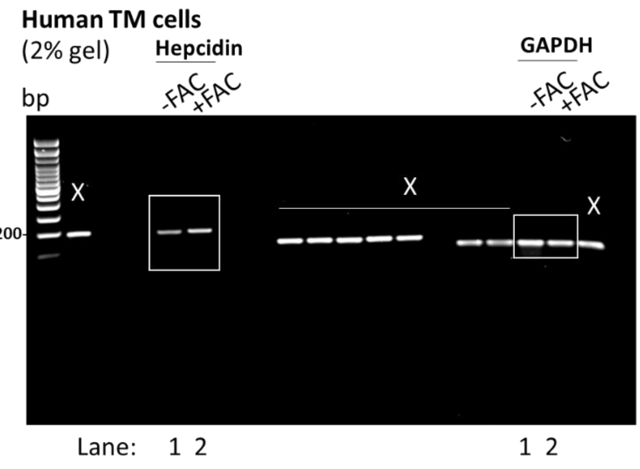

Legend: Full blots for Fig. 1.

Fig. 2 in manuscript

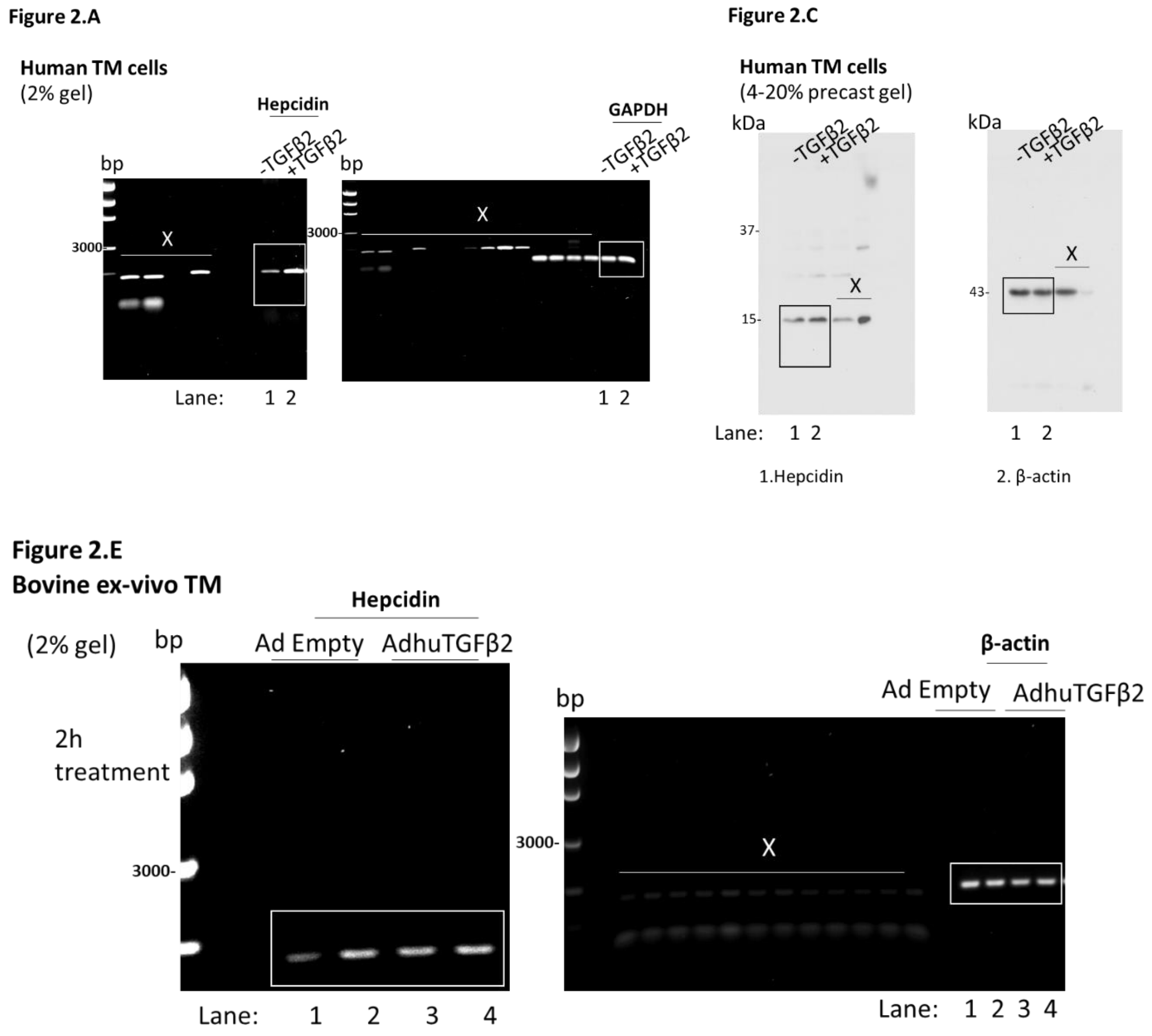

Figure 2.G

Bovine ex-vivo (12% gel)

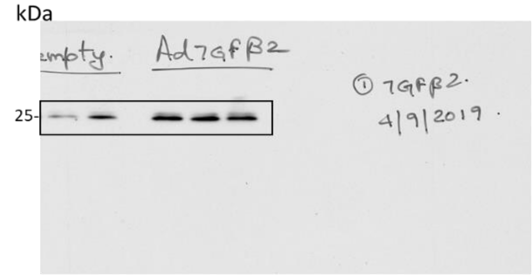

Lane: 1 2 3 4 5

1. TGFβ2

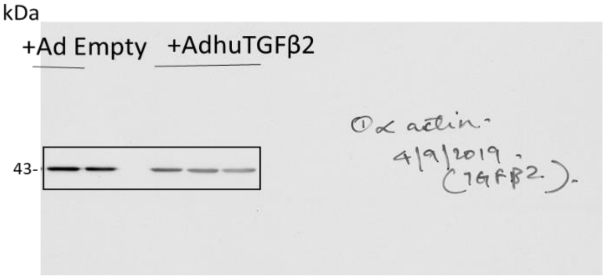

Lane: 1 2 3 4 5

2. β-actin

Bovine ex-vivo (4-20% precast gel)

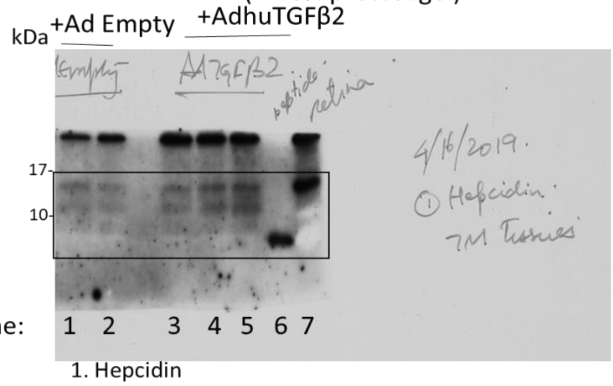

1. Hepcidin

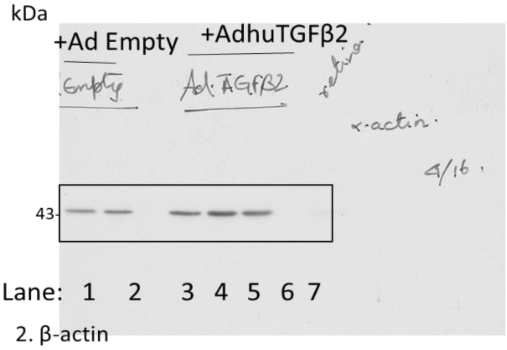

2. β-actin

Legend: Full blots for Fig. 2.

Fig. 3 in manuscript

Figure 3.A
Hu TM cells (12% gel)

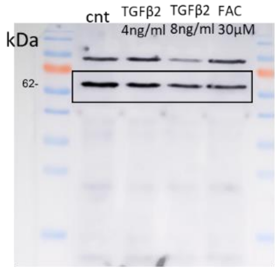

Lane: 1 2 3 4

1. Fpn

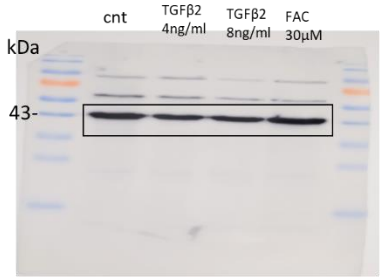

Lane: 1 2 3 4

2. β-actin

Legend: Full blots for Fig. 3.

Fig. 4 in manuscript

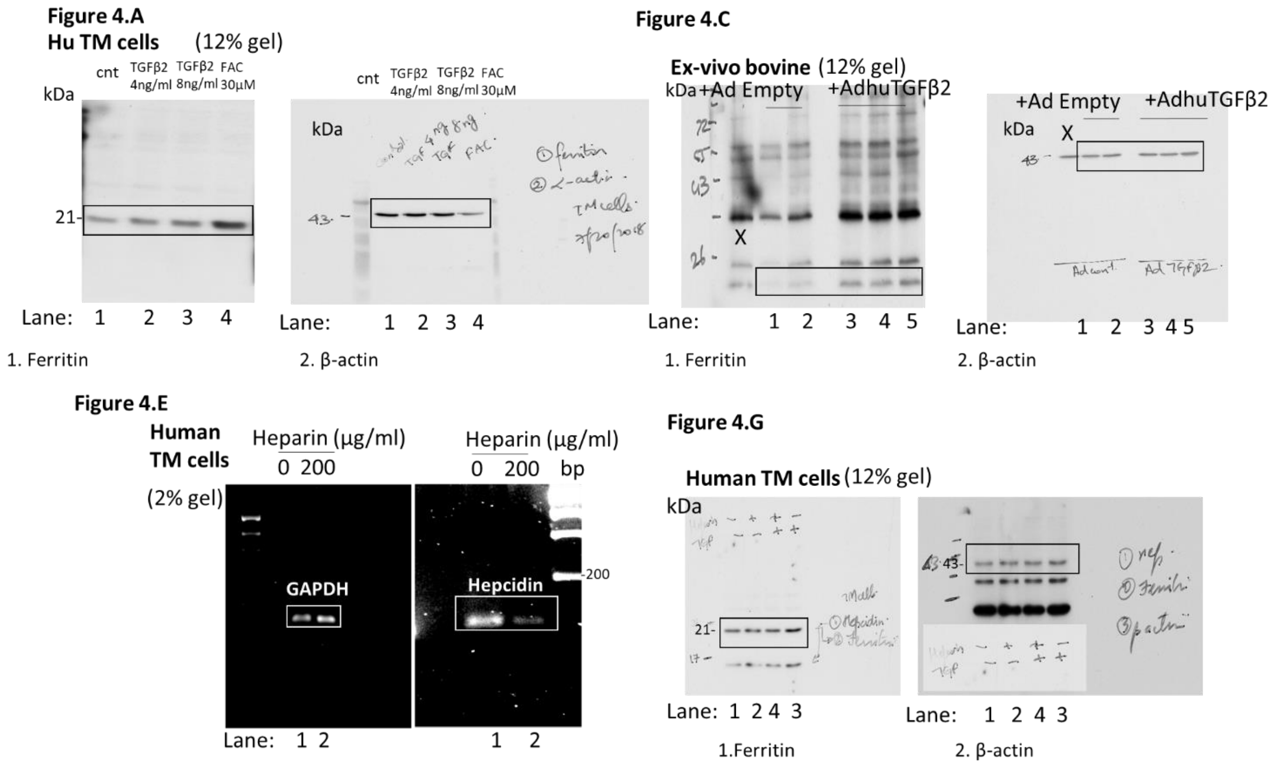

Legend: Full blots for Fig. 4.

Fig. 5 in manuscript

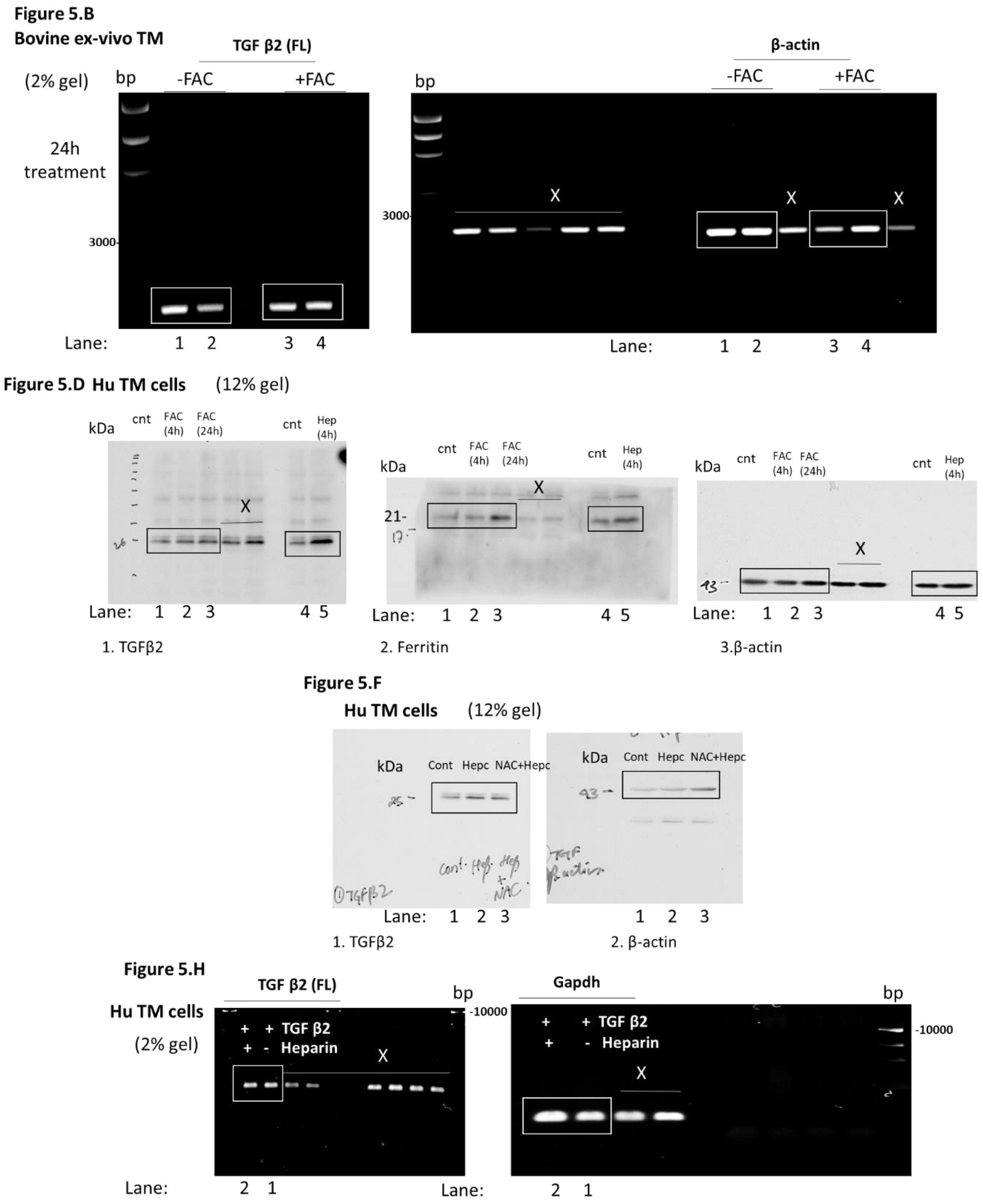

Legend: Full blots for Fig. 5.

X: marks all irrelevant samples

Supplementary Figure 2 (S2)

Fig S2.

The RT-PCR gel showed Hepcidin bands in the bovine TM tissues. The samples from the gel were processed and given to MCLAB DNA Sequencing Department to confirm if they were Hepcidin specific bands. The data received confirmed that the sequences were indeed bovine Hepcidin specific.

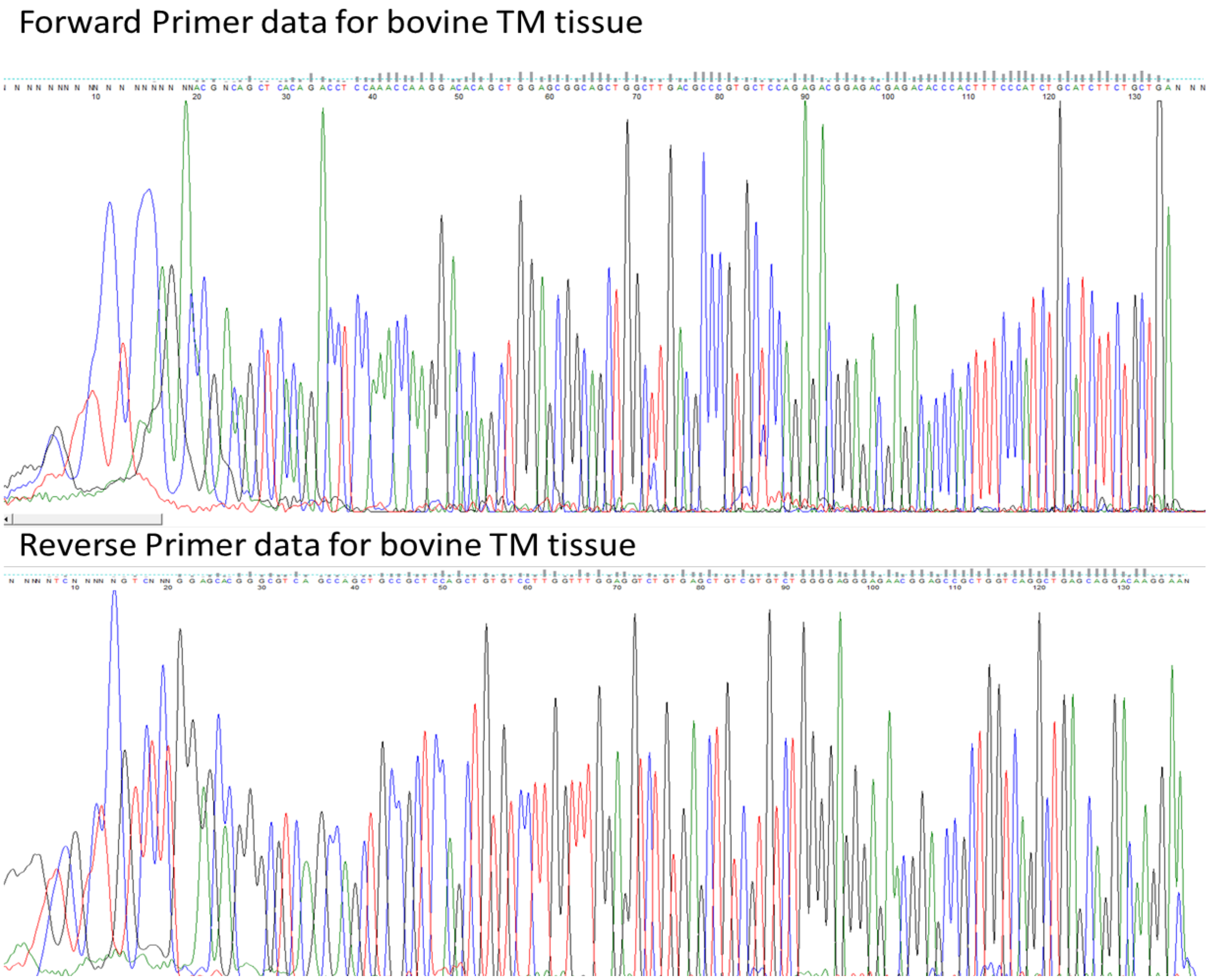

Legend Fig S2. The sequencing data when entered into the NIH BLAST software confirmed the primers to be bovine hepcidin specific thereby confirming the hepcidin specific band and the RT-PCR data.

Supplementary Figure 3 (S3)

Fig S3.

Different concentrations of TGFβ2 was tested on primary human TM cells to mimic glaucomatous alterations. 0,0.6 (physiological concentration), 4,8 and 10 ng/mL of TGFβ2 was treated for 48 hours and evaluated for expression of extra cellular matrix protein, vimentin which is known to overexpress confirming glaucomatous changes *in-vitro* **(Figure S3 A)**. An evident upregulation of vimentin with minimal toxicity (estimating the lactose dehydrogenase (LDH) content in the medium) was observed at 8ng/ml which was optimized for the study **(Figure S3 B & C)**. The Lactose Dehydrogenase (LDH) release assay was carried out to determine the cytotoxicity of different study treatments, by employing commercially available LDH detection kit (Roche-11644793001), as per manufacturer's instruction. Following treatments, the plate containing the cells was centrifuged at 1000 rpm for 10 min to obtain cell free supernatants, which were then incubated for 30 min in dark with the LDH substrate and the reading was obtained at 492 nm with reference wavelength of 620 nm using BioTek, Synergy 4 microplate reader.

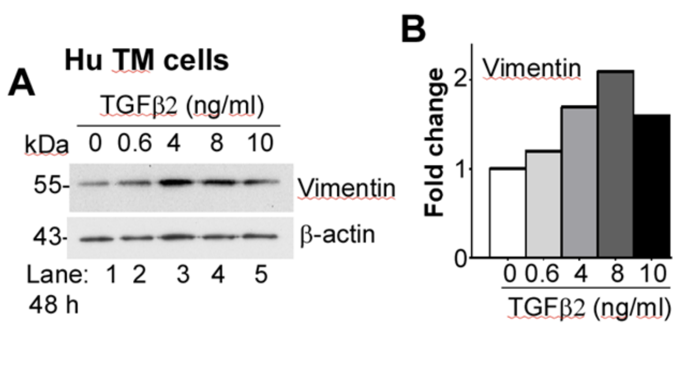

Legend Fig S3. (A) Immunoblotting results clearly show a marked change in vimentin expression following treatment with 8ng/ml for 48 h and hence this treatment concentration was

optimized for the experiments in the current study to mimic glaucoma in different models. **(B)** Density of protein bands after normalization with β-actin show a 2-fold increase of vimentin at 8ng/ml. **(C)** LDH assay show minimal toxicity at 0.6 (physiological concentration), 4 and 8ng/ml TGFβ2 concentration. However, at 10 ng/ml, there was a significant 27% cytotoxicity and hence 8ng/ml which showed highest vimentin expression and lowest cytotoxicity was considered as the optimal treatment to induce glaucomatous changes in primary human TM cells in-vitro with minimal cytotoxicity.

Supplementary Figure 4 (S4)

Fig S4.

Ex-vivo cultures of human anterior segment were transfected with AdhuTGFβ2 or AdEmpty control, and the change in pressure was monitored over time. The pressure increased significantly in AdhuTGFβ2-transfected sample relative to the control after 6 days.

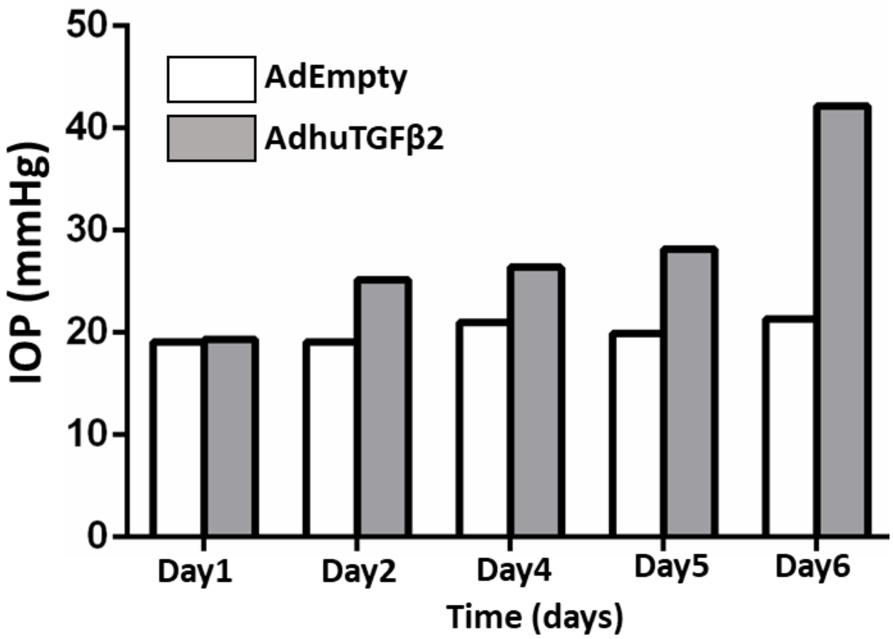

Legend Fig S4. Over expression of TGFβ2 with AdhuTGFβ2 in *ex-vivo* perfusion model of human anterior segment showed an increase in intraocular pressure (IOP) from 19 to 42 mmHg after 6 days. A parallel sample transfected with AdEmpty did not show any change. The experiment was done thrice with similar results and the representative data has been shown.

Supplementary Figure 5 (S5)

Fig S5.

Immunoblotting was carried out to verify if expression of ferritin in primary human trabecular meshwork cells responded to ferroportin change. Primary human trabecular meshwork cells were transfected with Ferroportin siRNA and scrambled controls (Dharmacon Inc.). Following ferroportin siRNA transfection, ferroportin expression downregulated and ferritin expression upregulated **(Figure S5 A)** in these cells. These data clearly indicate a positive response of iron levels following ferroportin alteration in TM cells. **Figure S5 B** shows the full blots.

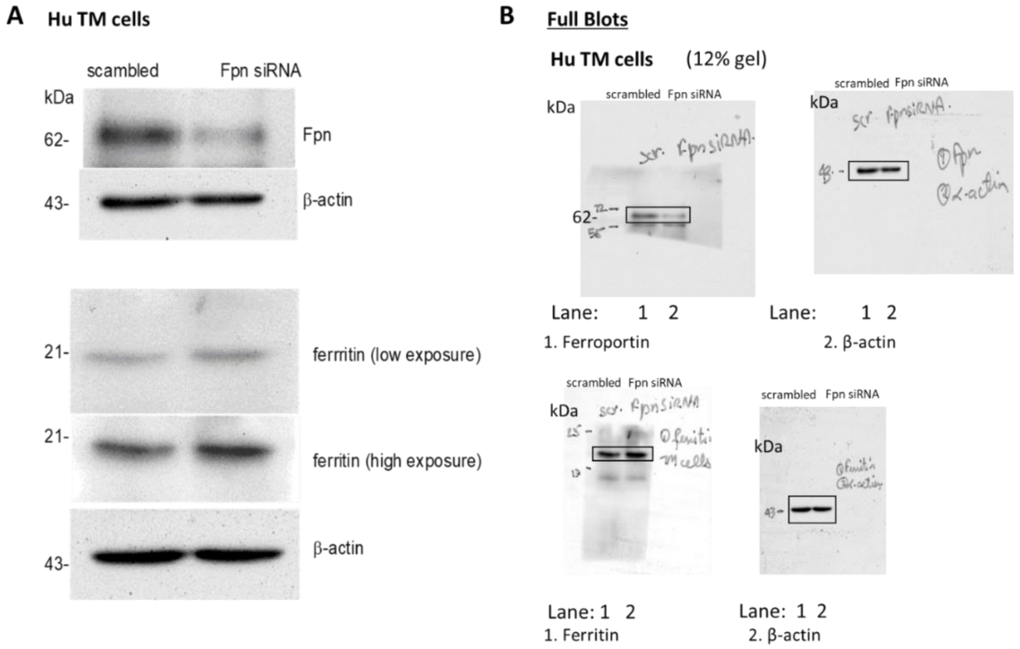

Legend Fig S5. (A) Immunoblotting was carried out for primary human TM cells treated with scrambled control and ferroportin siRNA. Ferroportin downregulated in the siRNA treated cells relative to the scrambled controls and there was an evident upregulation of ferritin. **(B)** Full blots are presented.

Supplementary Figure 6 (S6)

Fig S6.

Standardization of heparin concentration for effective hepcidin downregulation in primary human TM cells was carried out and the result is provided in Figure 4E, where heparin at 200µg/ml for 16 h proved most effective in blocking hepcidin. Heparin at 40-200µg/ml for 16 h did not induce significant cytotoxicity in primary human TM cells.

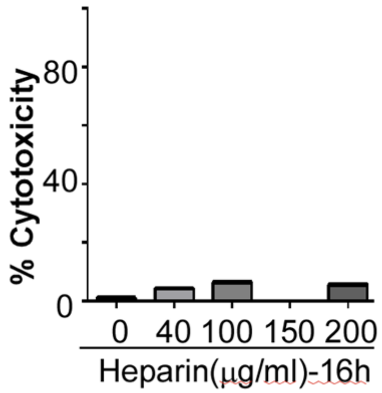

Legend Fig S6. LDH estimation was carried out for all heparin concentrations. No significant cytotoxicity was observed in any concentration of heparin treatments on primary human TM cells.

Supplementary Figure 7 (S7)

Fig S7.

Immunostaining of fixed sections, showed relatively stronger reactivity for ferritin in TM sections infected with AdhuTGFβ2 relative to AdEmpty controls **(Figure S7, panels 1-4)**. A serial section reacted with rabbit IgG control showed negative results, confirming the specificity of the reaction **(Figure S7, panel 5 & 6)**.

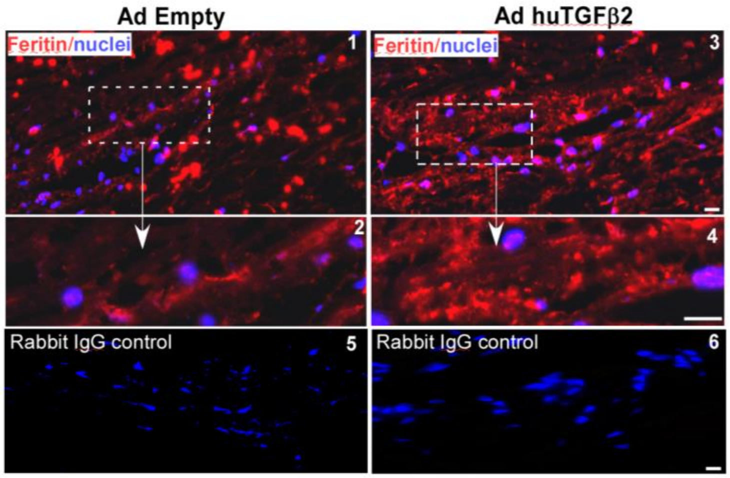

Legend Fig S7. Immunoreaction of fixed tissues from human anterior segment *ex-vivo* perfusion model shown in Figure 3C for ferritin shows more reactivity in samples over expressing TGFβ2 relative to controls (panels 3 & 4 vs. 1 & 2). Control sections reacted with rabbit IgG and processed in parallel did not show a positive signal (panel 5 & 6). Scale bar: 25 µm.

Supplementary Figure 8 (S8)

Fig S8.

H_2O_2 at a concentration of 0, 0.1, 0.2, 0.5, 1 and 2 mM was tested on primary human TM cells to optimize the concentration that induces maximum ROS generation with minimal cell death over 24 h. The experiment was conducted to standardize the positive control for DHE estimation in these cells (superoxide anion) following different study treatments. LDH assay using the supernatant as per kit instructions and ROS estimation (DHE staining) using the corresponding primary human TM cells clearly show an evident ROS generation of 20% at 0.2 mM H_2O_2 with minimal cytotoxicity (2%). Therefore, this concentration was optimized as the positive control for all ROS estimation experiments in the current study.

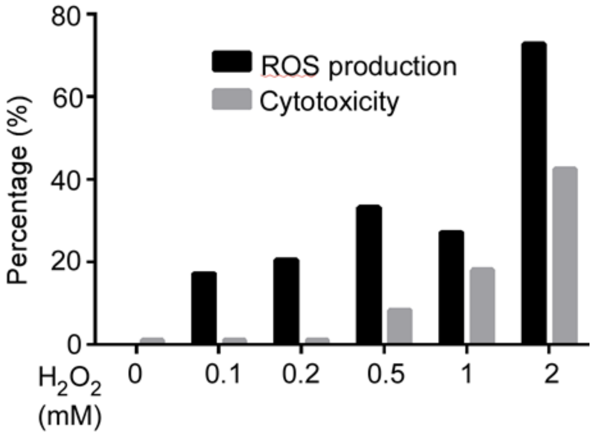

Legend Fig S8. Different H_2O_2 concentrations were tested on primary human TM cells to estimate the optimal positive control for ROS estimation using DHE staining in these cells. 0.2 mM of H_2O_2 for 24 h induced ROS production with minimal toxicity. Hence H_2O_2 at 0.2 mM was used as the positive control for ROS estimation using DHE staining in the study.

Supplementary Figure 9 (S9)

Fig S9.

LDH estimation was carried out to calculate cytotoxicity in the treatments in Figure 5A. None of the study treatments showed significant cytotoxicity.

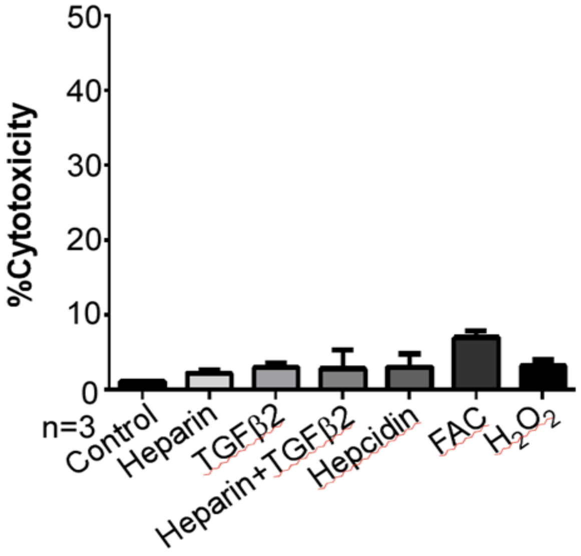

Legend Fig S9. LDH estimation was carried out for treatments in Figure 5A using cell supernatant. None of the treatment media showed a significant increase in LDH concentration relative to the control indicating no significant cytotoxicity.
